# The ground beetles (Coleoptera, Carabidae) of southern Kuril Islands, Russia

**DOI:** 10.3897/BDJ.9.e75529

**Published:** 2021-11-17

**Authors:** Kirill V Makarov, Yuri N Sundukov

**Affiliations:** 1 Moscow State Pedagogical University, Institute of Biology & Chemistry, Zoology & Ecology Department, Moscow, Russia Moscow State Pedagogical University, Institute of Biology & Chemistry, Zoology & Ecology Department Moscow Russia; 2 Federal Scientific Center of the East Asia Terrestrial Biodiversity, Far East Branch of the Russian Academy of Sciences, Vladivostok, Russia Federal Scientific Center of the East Asia Terrestrial Biodiversity, Far East Branch of the Russian Academy of Sciences Vladivostok Russia

**Keywords:** Far East, insular fauna, occurrence, species richness, local fauna

## Abstract

**Background:**

We compiled a list of the ground beetles that have been confirmed to occur to date in the southern Kuril Islands, Russian Far East. The list includes 168 species, all of which are known from Kunashir Island and the species richness on the remaining islands ranges from 68 (Shikotan Island) to 21 species (Tanfil'eva Island). The species richness is shown to depend sublinearly on island area, this being unusual for island faunas (Triantis et al. 2011).

**New information:**

A large part of data is published here for the first time on the records of ground beetles in the southern Kuril Islands with precise localities. This allows not only the taxonomic composition of the faunas, but also the composition of local faunas to be discussed.

## Introduction

Starting with the paper by Kuwayama (1967), who listed 20 species of ground beetles in Kunashir Island, about fifteen studies containing information on the fauna of the islands have since been published. For example, Krivolutskaja (1973) listed 66 carabid species in Kunashir and 75 for the southern Kuril Islands in her monograph on the insects of the entire Kuril Archipelago, while Kryzhanovskij et al. (1975) recorded already 113 species.

Later, the data on the ground beetles of Kunashir were replenished even more. For example, the research of G. Sh. Lafer (Lafer 1989, Lafer 1992, Lafer 1996) increased the number of ground beetle species to 140, whereas as a result of a series of subsequent papers (Sundukov 2001, Sundukov 2008, Makarov and Sundukov 2011, Sundukov 2011, Sundukov 2013, Makarov and Sundukov 2014, Sundukov and Makarov 2021), already 158 species have been confirmed in the Island’s fauna. Moreover, we predicted (Sundukov and Makarov 2016) that at least 170 ground beetle species could be found on Kunashir.

The Global Biodiversity Information Facility (GBIF) repository, aside from the general list of animal species of Kunashir (Kozlovsky et al. 2020) comprising 157 species of ground beetles (applying the modern understanding of a number of taxa), contained no information on the composition and distribution of the carabid fauna of the southern Kuril Islands.

The body of information accumulated so far makes it possible to discuss both the differences in the faunas of the individual islands studied and the heterogeneity of the species composition within Kunashir as the largest island.

## Project description

### Title

Carabidae of South Kuriles

### Personnel

Yuri Sundukov, Kirill Makarov

## Sampling methods

### Study extent

**Figure 1.**
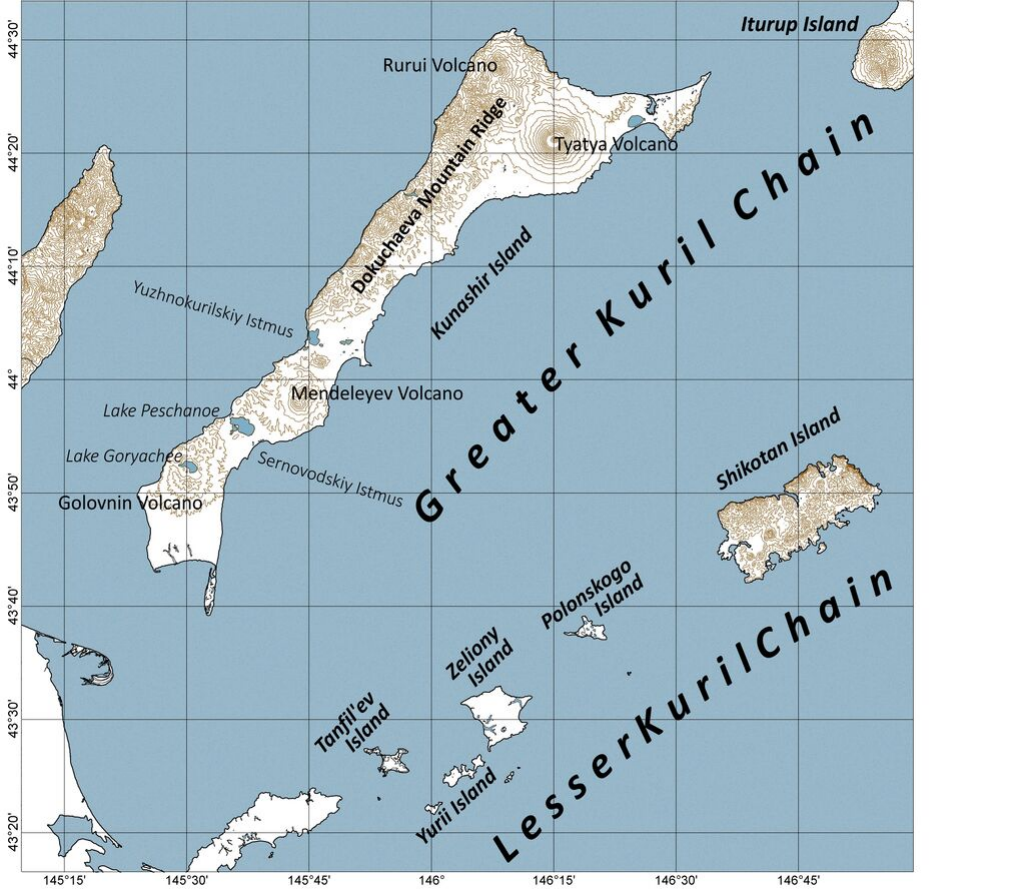
Study area of the Kuril Islands.

The studies were conducted on the southern islands of the Kuril Archipelago within the boundaries of the Yuzhno-Kurilsky administrative district of the Sakhalin Oblast. The district includes the Kunashir Island of the Greater Kuril Chain and all islands of the Lesser Kuril Chain (Fig. 1). Most of the islands studied are parts of the State Nature Reserve "Kurilskiy" and the State Nature Reserve "Malye Kurily".

The climate of the southern Kuril Islands is moderate humid maritime, with a strong influence of the Sea of Okhotsk and the Pacific Ocean. The Islands are characterised by a harsh wind regime (with gusts up to 35-50 m/s), high rates of precipitation (1200–1500 mm per year), relatively mild winters and cool summers (average temperature of the coldest month is -5.6°C and of the warmest month, +15.5°C). Waters of the warm Soya current approach the Okhotsk coast of the Archipelago and the cold Kuril-Kamchatka (Oyashio) current approaches the Archipelago from the Pacific side.

Kunashir is the southernmost and one of the largest islands of the Great Kuril Chain. The terrain of the Island is mainly volcanic and it consists of three mountain ranges formed by four active volcanoes: in the northern part, the isolated Tyatya Volcano (1819 m a.s.l.) and the Ruruy Volcano (1485 m a.s.l.) as the highest place of the Dokuchaeva Mountain Ridge; in the central part, the Mendeleev Volcano (886 m a.s.l.); and the Golovnin Volcano (541 m a.s.l.) in the southern part. The mountain ranges are separated by isthmuses: Yuzhnokurilskiy and Sernovodskiy, which are composed of marine sediments and volcanic folded Neogene rocks. Kunashir, along with Iturup, has the greatest landscape diversity amongst the islands of the Kuril Archipelago. The dense river network is formed by numerous mountain and lowland rivers and streams, many of which have high temperatures and mineralisation content. Amongst the two dozen lakes on the Island, the largest freshwater lake in the Kuril Islands is Lake Peschanoe, while the largest thermal lake is caldera Lake Goryachee.

The vegetation of Kunashir is noticeably richer and more diverse than on the other islands of the Archipelago (Figs 2, 3). According to V. Yu. Barkalov (Barkalov 2009), 1087 species of vascular plants grow on the Island, these accounting for almost 80% of the Tracheophyta species recorded on the Archipelago. Dark coniferous (Fig. 2d), Erman's birch (Fig. 2c) and mixed coniferous-broadleaved forests (Fig. 2e, f) are widespread on the Island. The river floodplains are taken up by alder-birch forests (Fig. 3a) and thickets of coastal willows (Fig. 3b). Grass-sedge meadows (Fig. 3c) and moss bogs (Fig. 3f) are common in the lower reaches and estuaries of rivers; while dry herb and dwarf bamboo meadows (Fig. 3e) on sandy and ocherous soils are common on the sea coast.

Shikotan is the northernmost and largest island of the Lesser Kuril Chain. Its terrain is formed by steep hills and low-mountain massifs, the highest places being the Shikotan (412 m a.s.l.), Ploskaya (363 m a.s.l.), Notori (357 ml a.s.l.) and Tomari (356 m a.s.l.) mountains. The hydrographic network is quite dense and consists of small freshwater mountain rivers and streams. There are neither lakes nor thermal springs. A distinctive feature of the Island is the absence of altitudinal zonation, the vegetation thus being represented by a mosaic of dwarf bamboo meadows (Fig. 4b), small dark coniferous-birch forests (Fig. 4a) and elevated dwarf shrub bogs (Fig. 4d). Alder forests (Fig. 4c), thickets of coastal willows (Fig. 4e) and swamp grass-sedge meadows (Fig. 4f) are widespread in the river floodplains.

Polonskogo Island is located 25 km south of Shikotan. The coastline is weakly indented by small bays. The surface of the Island is low and flat, at no point exceeding 16 m a.s.l. The shores are occupied by sand and pebble beaches or eroded peat bogs approaching the water. There are no rivers, but only short streams with narrow depressed channels and swampy banks and two rather large freshwater lakes. The Island is completely devoid of forest vegetation. Its elevated shore banks are covered with dense herb meadows and wild rose thickets (Fig. 5a), while the central part and floodplains of streams are covered with sedge, reed or sedge-moss bogs (Fig. 5b).

Yurii Island is located in the south of the Lesser Kuril Chain. The coastline is heavily indented, with deep bays over the entire extent of the western coast. The shores are mostly rocky. The terrain is formed by four undulating land massifs connected by low isthmuses. The height of the watersheds ranges from 20 to 30 m a.s.l., the highest elevation being 44 m a.s.l. The isthmuses are occupied by low-level swamps and small lagoon lakes. There are sand beaches in the larger bays, while the remaining shore is occupied by pebble or large-block beaches. There are only small streams with narrow channels depressed into the clay soil and swampy banks. There is no forest vegetation, the upland areas being covered with dense herb meadows (Fig. 5c) and the lowlands with sedge-moss bogs (Fig. 5d).

Tanfil'eva Island is the southernmost island of the Lesser Kuril Chain, located 5 km off the north-eastern coast of Hokkaido. The landscape is flat, with the greatest elevations reaching up to 16 m a.s.l. The coastline is strongly indented, forming wide bays and headlands that protrude far into the sea. Short streams and several lagoon lakes, the largest of which are located near the east coast, represent the hydrographic network of the Island. The relief and vegetation are similar to those on Polonskogo Island (Fig. 5e, f).

### Sampling description

The present study was based on the material we collected on Kunashir, Shikotan, Polonskogo, Yurii and Tanfil’eva Islands in 1990, 2008, 2009 and 2011–2018, as well as the collections of the Federal Scientific Center of East Asia Terrestrial Biodiversity, the Far Eastern Branch of the Russian Academy of Science, Vladivostok, the Moscow Pedagogical State University, Moscow and the Zoological Institute of the Russian Academy of Sciences, Saint Petersburg.

**Figure 6.**
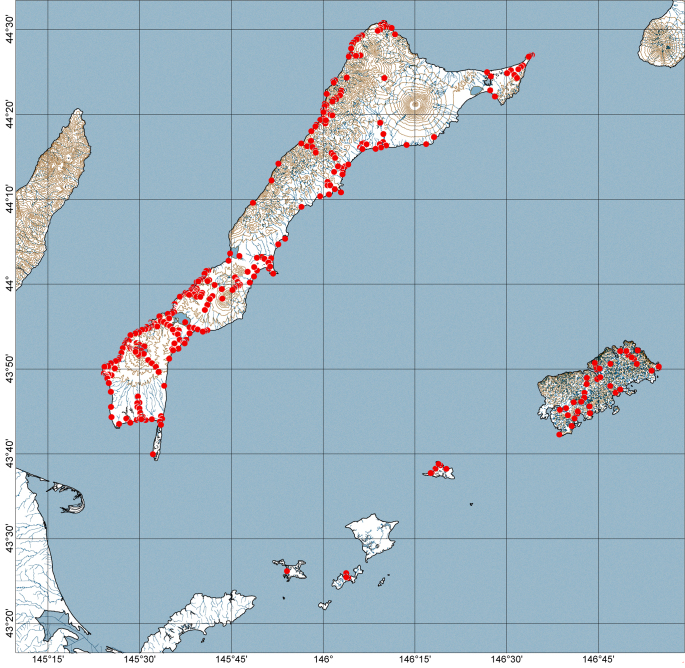
Ground beetle collecting localities

Most ground beetles were collected by hand; to a lesser extent by beating vegetation, sifting the litter and using window flight and soil traps.The places of capture of ground beetles are shown in the Fig. 6.

## Geographic coverage

### Description

Kunashir Island and the neighbouring islands of the Lesser Kuril Chain. To clarify the distribution of certain taxa, the table includes data on the records from the Sakhalin, Iturup and Urup Islands and mainland Russian Far East.

### Coordinates

43.341 and 44.559 Latitude; 145.377 and 147.09 Longitude.

## Taxonomic coverage

### Taxa included

**Table:** 

Rank	Scientific Name	Common Name
family	Carabidae	Ground beetles (EN), Жужелицы (RU)

## Traits coverage

Altogether, 168 carabid species are known to occur in the southern Kuril Islands, on the basis of both literature data and museum collections. We exclude four species (Table 1) from this list which are known from single records from Kunashir Island, as we later conducted large-scale surveys at the locations of those records, but obtained none of these species.

Thus, the richness of the ground beetle fauna of the southern Kuril Islands totals 168 species. All these species are known from Kunashir Island [there are records of five species of ground beetles from Shikotan Island which have not been found on Kunashir to date (Kryzhanovskij et al. 1975, Lafer 1989); however, none of those records has been confirmed by our material]. The faunas of ground beetles of the other Islands resemble versions of the fauna of Kunashir impoverished to varying degrees and ranging between 68 and 21 species (Table 2). We exclude data for the Zelenyi and Anuchin Islands from further analysis due to insufficient material.

**Figure 7.**
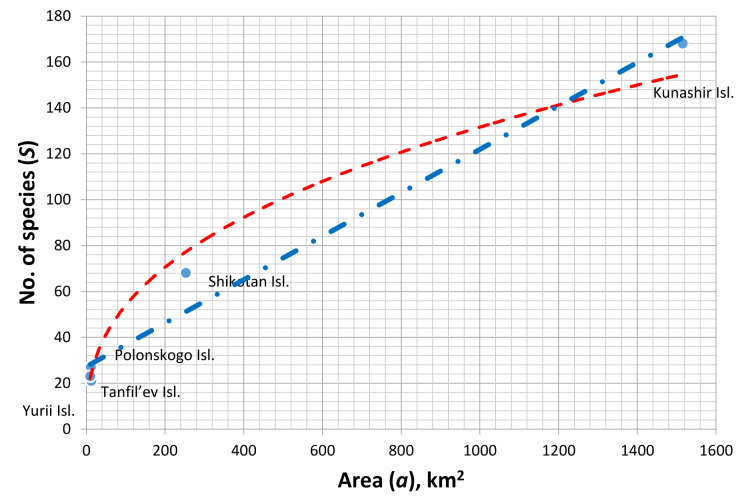
The species richness of the ground beetle fauna of the southern Kuril Islands depending on island area (red line - the power function *S* = 9.0968 *a*^0.9811^, blue line - the linear function *S* = 0.0933*a* + 27.373)

The number of species depending on island area (Fig. 7) is well approximated by the power function *S* = 9.0968 *a*^0.9811^ (R^2^ = 0.9811). However, as the power coefficient is close to 1, the dependence does not differ from the linear one which describes the observed pattern just as well (R^2^ = 0.9748).

The obtained parameters of the classical power dependence *S* = C*a*^Z^ (Sugihara 1981, Dengler 2009) are partially comparable with those known for the faunas of island beetles (Niemelä et al. 1987, Niemelä 1988, Browne and Peck 1996, Kotze et al. 2000, Fattorini 2002, Zalewski and Ulrich 2006, Trichas et al. 2008): C = 9.0968 (the cited publications report on values ranging from 0.525 to 11.321). However, the power coefficient Z = 0.9811 is significantly outside the range (0.06–0.449) indicated in the literature sources. Earlier, high Z values were suggested to indirectly indicate a large role of extinction processes (Fattorini and Borges 2012) in the formation of island beetle faunas. This is consistent to some extent with the history of Kuril Archipelago: the more ancient islands of the Lesser Kuril Chain are gradually decreasing in area under the impact of multidirectional tectonic processes (Razzhigaeva et al. 2009) and erosion (Markov 2009).

As we demonstrated earlier, the beetles of the Kunashir Island form at least two local faunas, "northern" and "southern", the border between both roughly corresponding to the Yuzhnokurilskiy isthmus (Makarov et al. 2013).

The features of the "northern" local fauna are determined by the endemic *Bembidionruruy* Makarov et Sundukov, 2014, which we consider a rare case of a Pleistocene endemic to the Kunashir fauna (Makarov and Sundukov 2014), as well as a number of ground beetle species known only from the Dokuchaeva Mountain Ridge or its spurs: *Trechusnakaguroi* Uéno, 1960, *Bembidionlucillumlucillum* Bates, 1883, *Diploussibiricusatratus* Habu, 1951 and *D.depressus* (Gebler, 1829). In addition, the Dokuchaeva Ridge is the only habitat for a number of vertebrate and insect species from different orders on Kunashir Island (Sundukov and Makarov 2016).

The fauna of the "southern" block is more heterogeneous. On the one hand, the endemic subspecies *Cylinderaelisae* (Motschulsky, 1859) and *Bembidionsanatum* Bates, 1883 are known from the Mendeleev Volcano. On the other hand, the peculiarity of this fauna is determined by the species found in the very south of the Island and widespread in Japan (*Bembidionyokahamae* (Bates, 1883), *Amarachalcophaea* Bates, 1873), for which a recent (and possibly repeated) penetration into the Island seems to be most likely.

### Data coverage of traits

The dataset (Makarov and Sundukov 2021) includes finds of 168 species on five islands of the Kuril Archipelago - a total of 1320 locations

## Temporal coverage

### Notes

1990, 2008, 2009, and 2011–2018 years

## Collection data

### Collection name

DUBC — Daugavpils University Beetles collection (Ilgas, Latvia); FEB - Federal Scientific Center of the East Asia Terrestrial Biodiversity FEB RAS (Vladivostok, Russia); MPU - Moscow State Pedagogical University (Moscow, Russia); SIEE - Institute of Ecology and Evolution. A.N. Severtsov RAS (Moscow, Russia); VNIIKR — All-Russian Plant Quarantine Center, (Bykovo, Moscow Region, Russia); ZIN - Zoological Institute RAS, (St. Petersburg, Russia)

### Specimen preservation method

Dried

## Usage licence

### Usage licence

Creative Commons Public Domain Waiver (CC-Zero)

### IP rights notes

This work is licensed under a Creative Commons Attribution (CC-BY) 4.0 License.

## Data resources

### Data package title

Carabidae of South Kuriles

### Resource link

https://www.gbif.org/dataset/ec534611-51bc-48f7-867e-f0e6500fb2a8

### Alternative identifiers

http://gbif.ru:8080/ipt/resource?r=skurilescarabidae

### Number of data sets

1

### Data set 1.

#### Data set name

Carabidae of South Kuriles

#### Data format

Darwin Core

#### Number of columns

36

#### Character set

UTF-8

#### Download URL

https://www.gbif.org/occurrence/download?dataset_key=ec534611-51bc-48f7-867e-f0e6500fb2a8

#### Data format version

1.4

#### Description

Carabidae (Coleoptera) of South Kuriles: Kunashir Island and Lesser Kuriles, Iturup and Sakhalin Islands partim.

**Data set 1.** 

Column label	Column description
OccurenceID	Simple identifier - prefix "CSK" and ascending number
scientificName	The full scientific name, including author and year
kingdom	Animalia (in all records)
phylum	Arthropoda (in all records)
class	Insecta (in all records)
order	Coleoptera (in all records)
family	Full scientific name of the family in which the taxon is classified (Carabidae, in all records)
genus	Generic name
specificEpithet	The name of the first or species epithet of the scientificName
infraspecificEpithet	The name of the last or species epithet of the scientificName
taxonRank	The taxonomic rank of the most specific name in the scientificName (species or subspecies)
organismQuantity	A number value for the quantity of specimens
organismQuantityType	The type of quantification system used for the quantity of organism
verbatimLocality	The original textual description of the place
verbatimCoordinates	The verbatim original spatial coordinates
verbatimEventDate	The verbatim original representation of the date information
recordedBy	A person,responsible for recording the original Occurrence
basisOfRecord	Preserved Specimen (in all tables)
continent	Asia (in all records)
country	Russia (in most records)
countryCode	Country code
stateProvince	Sakhalinskaya Oblast', in most records
islandGroup	The name of the island group in which the Location occurs
island	The name of the island on or near which the Location occurs
locality	The specific description of the place
habitat	Category or characteristic of the habitat in which the beetles are collected
eventDate	The date or interval during which an Event occurred
year	The four-digit year
month	The integer month
day	The integer day
verbatimCoordinateSystem	In all tables: degrees, minutes, seconds
geodeticDatum	Geodetic datum, WGS84 in all records
decimalLatitude	The geographic latitude
decimalLongitude	The geographic longitude
coordinateUncertaintyInMetres	The horizontal distance (in metres) from the given decimalLatitude and decimalLongitude describing the smallest circle containing the whole of the Location
institutionCode	The acronym in use by the institution having custody of the object(s) or information referred to in the record (DUBC, FEB, MPU, SIEE, VNIIKR, ZIN)

## Figures and Tables

**Figure 2a.**
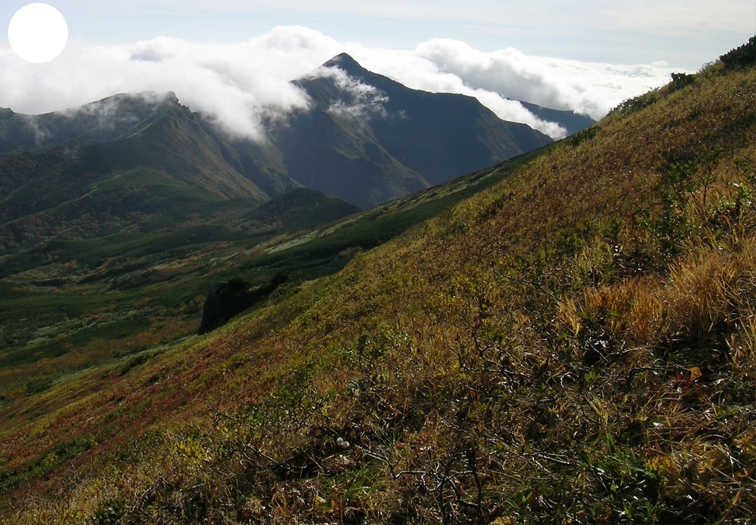
subalpine vegetation

**Figure 2b.**
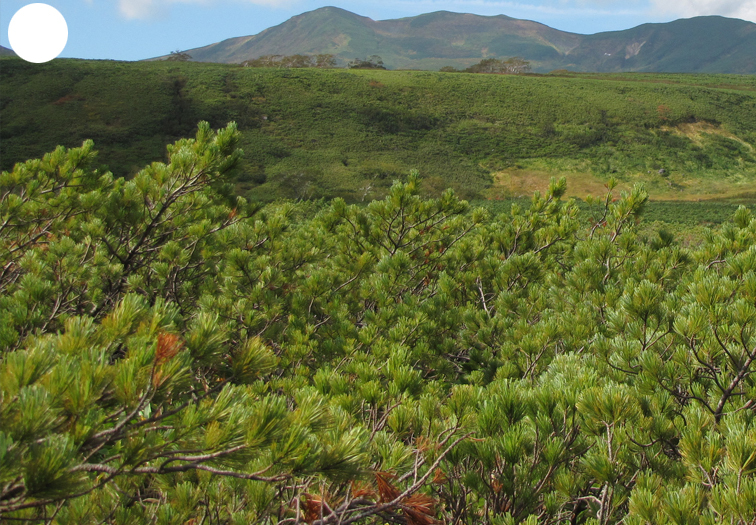
dwarf cedar forest

**Figure 2c.**
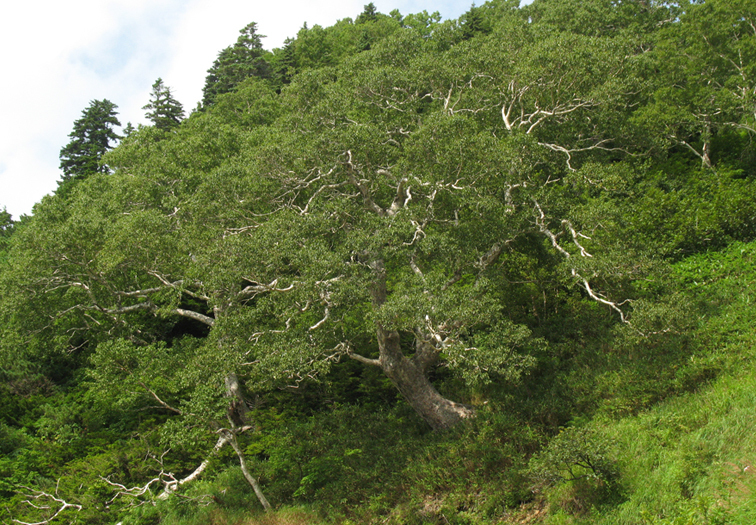
Erman's birch forest

**Figure 2d.**
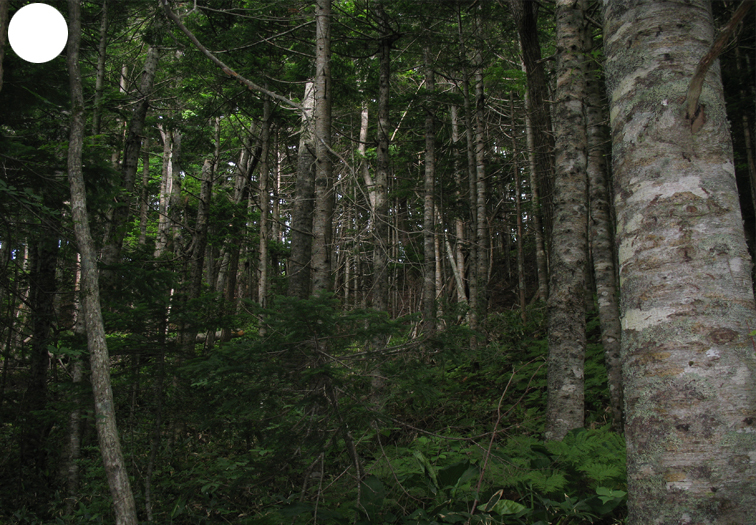
dark coniferous forest

**Figure 2e.**
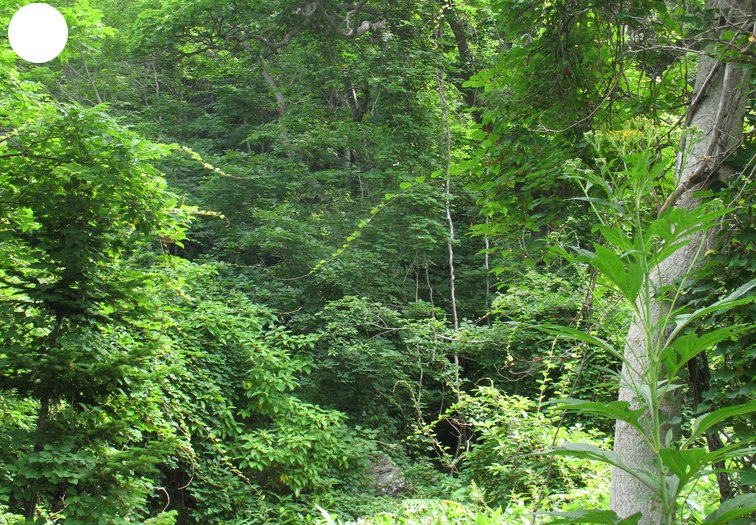
mixed coniferous-broadleaved forests

**Figure 2f.**
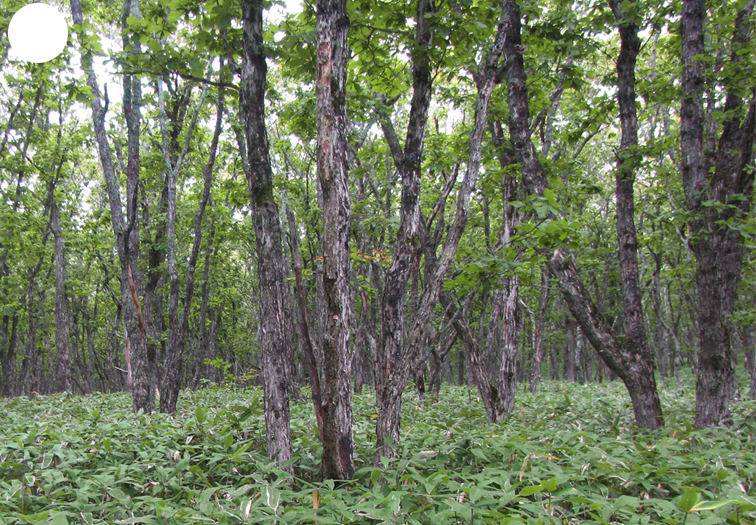
broadleaved forests

**Figure 3a.**
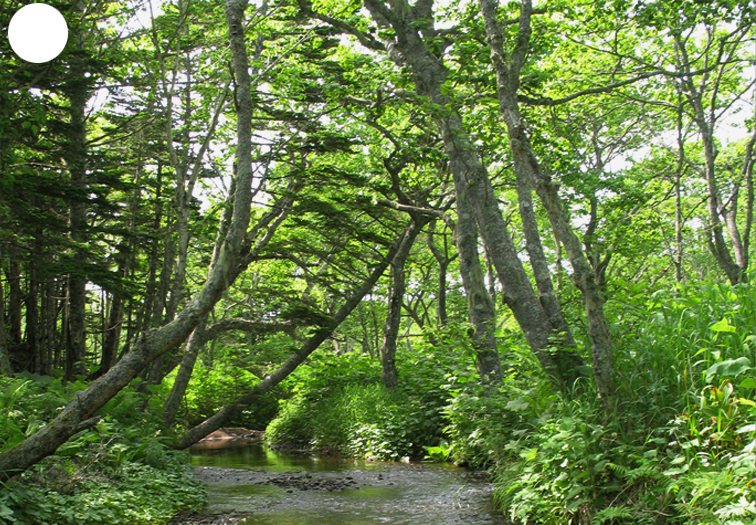
alder-birch forest

**Figure 3b.**
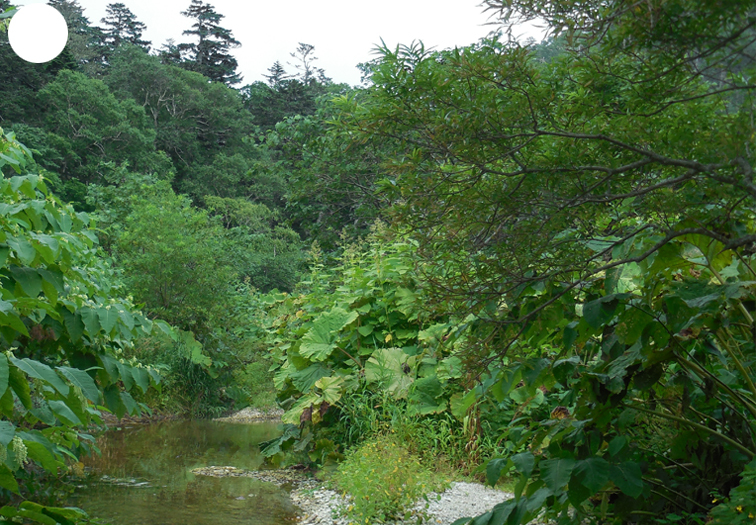
coastal willow

**Figure 3c.**
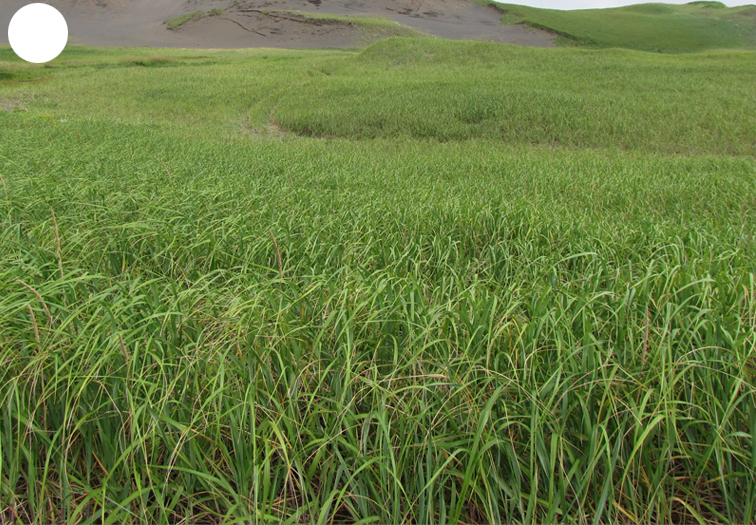
grass-sedge meadow

**Figure 3d.**
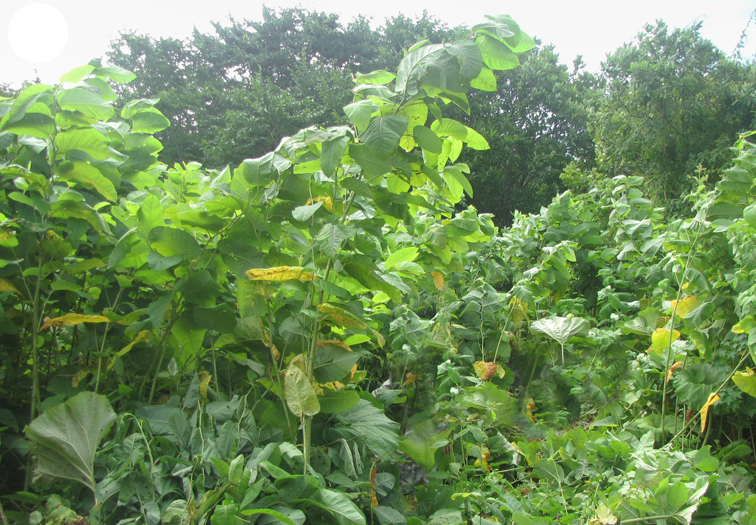
tall grass

**Figure 3e.**
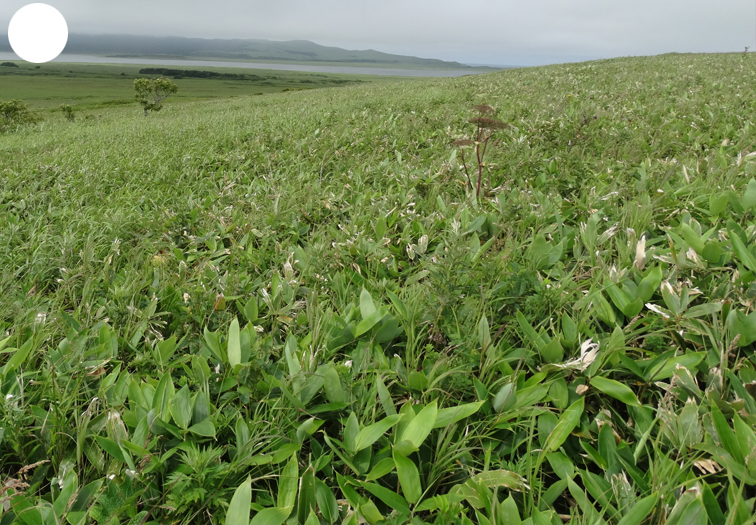
dwarf bamboo meadow

**Figure 3f.**
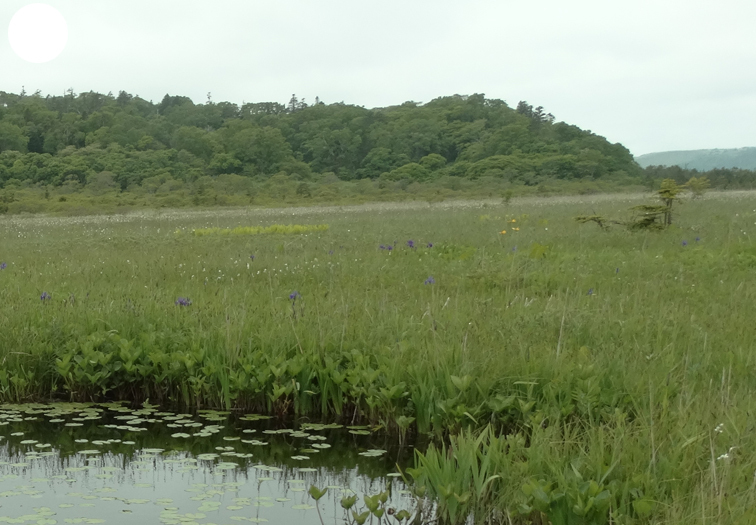
sedge-moss swamp

**Figure 4a.**
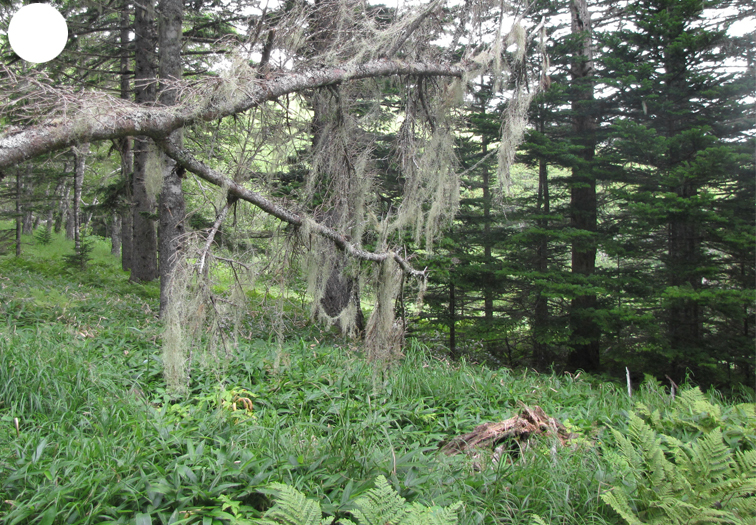
dark coniferous-birch forest

**Figure 4b.**
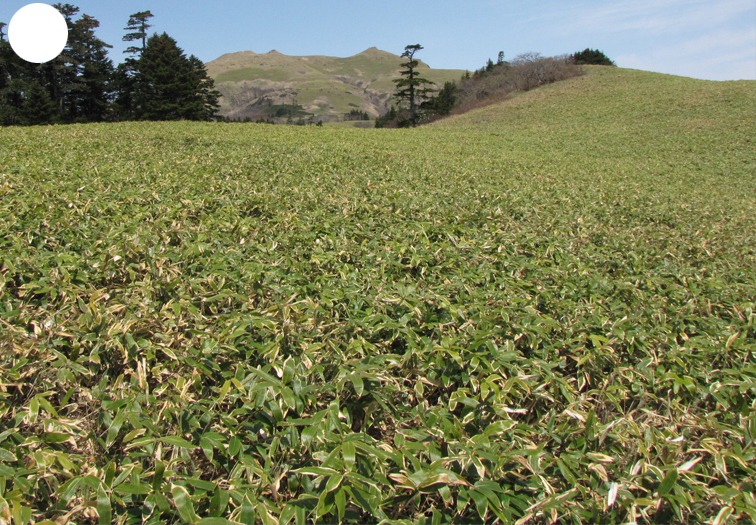
dwarf bamboo meadow

**Figure 4c.**
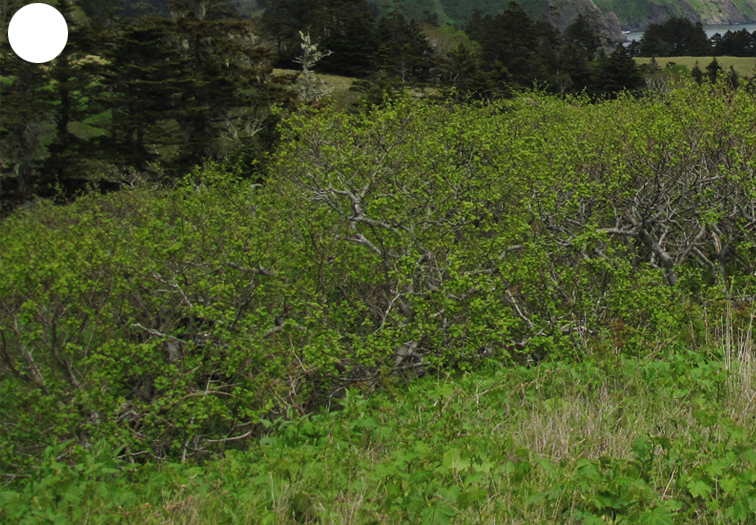
alder forest

**Figure 4d.**
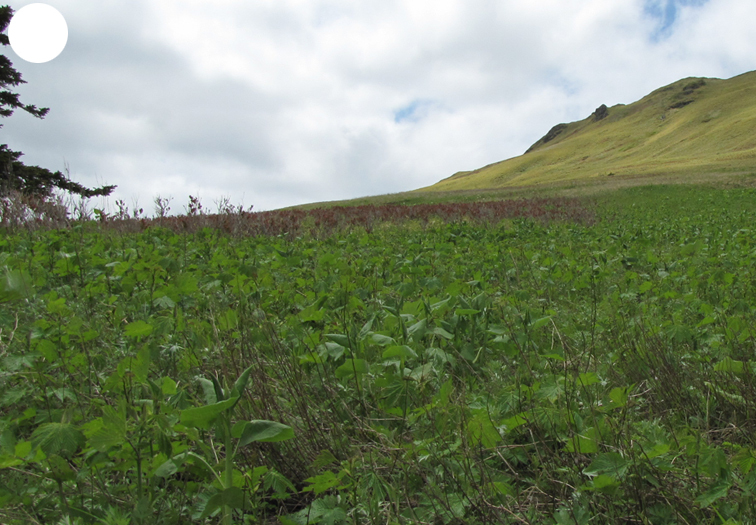
elevated dwarf shrub bog

**Figure 4e.**
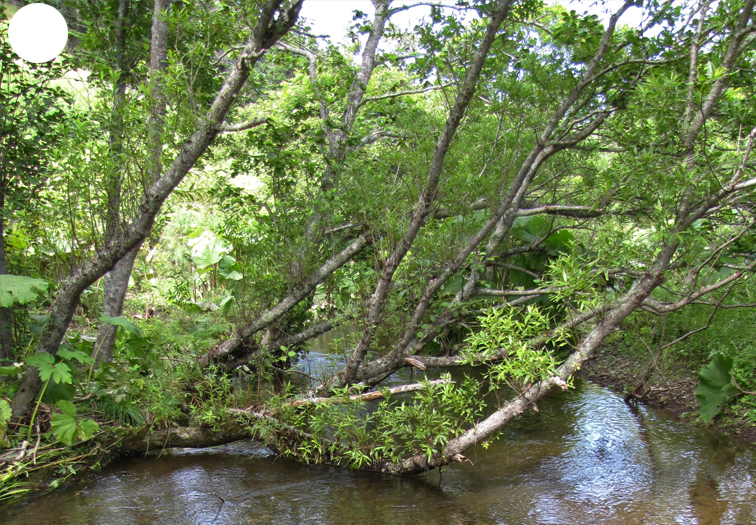
coastal willow

**Figure 4f.**
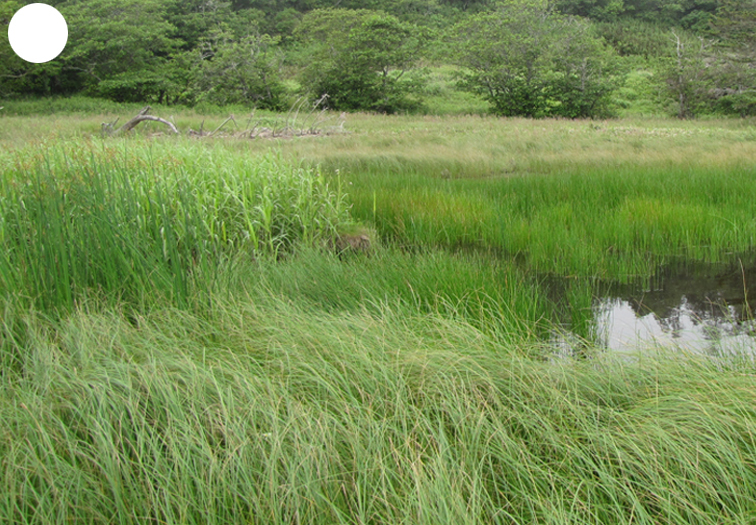
swamp grass-sedge meadow

**Figure 5a.**
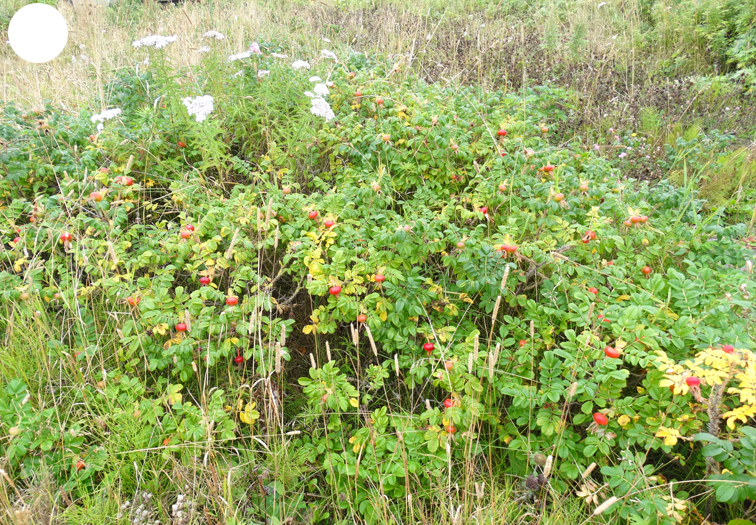
Polonskogo Island, herb meadow and wild rose thickets

**Figure 5b.**
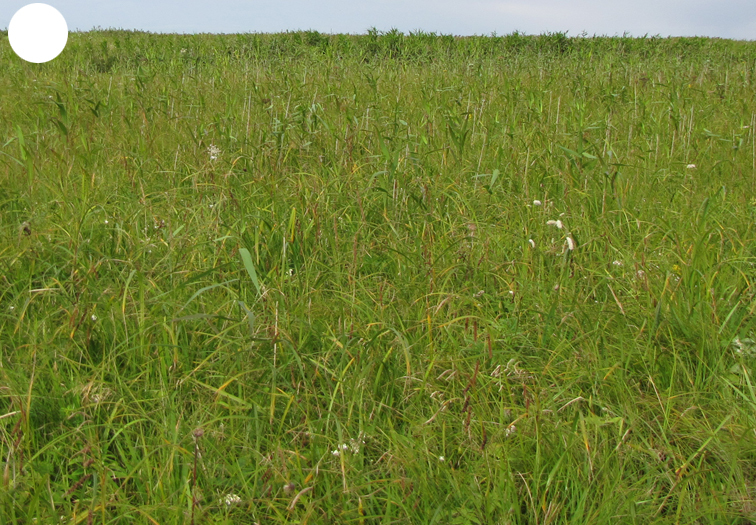
Polonskogo Island: reed bog

**Figure 5c.**
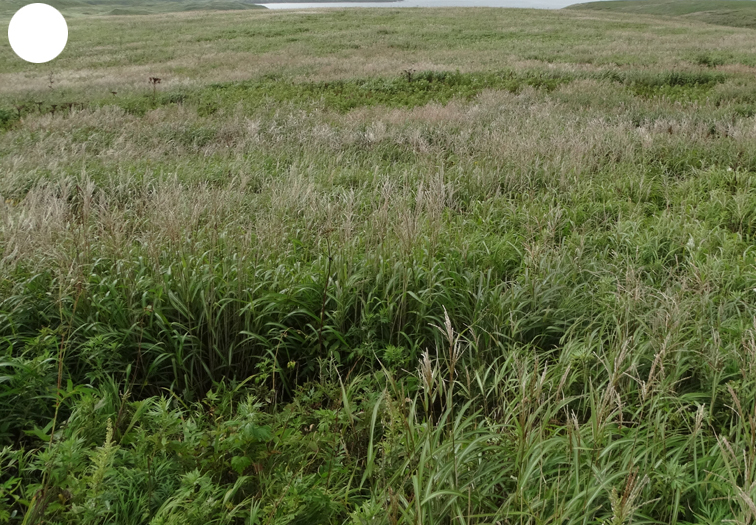
Yurii Island, dense herb meadow

**Figure 5d.**
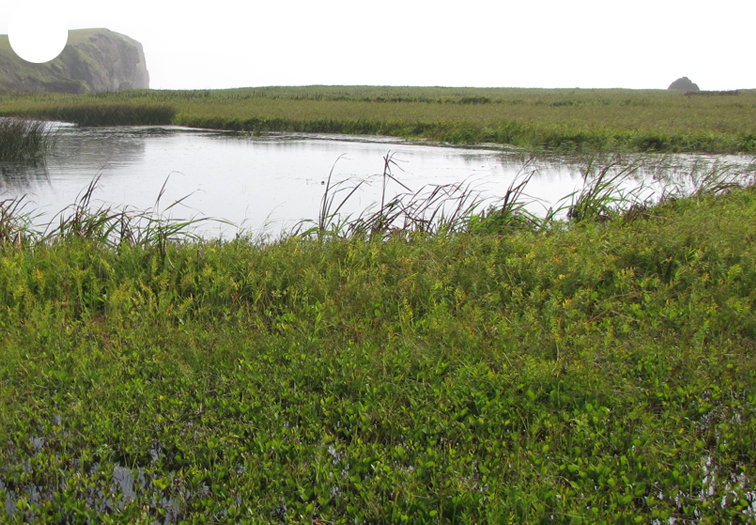
Yurii Island, sedge-moss bog

**Figure 5e.**
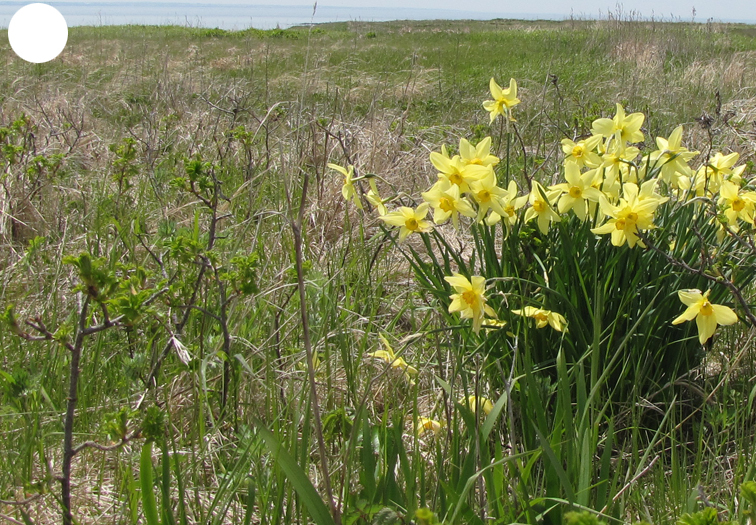
Tanfil'ev Island, dense herb meadow

**Figure 5f.**
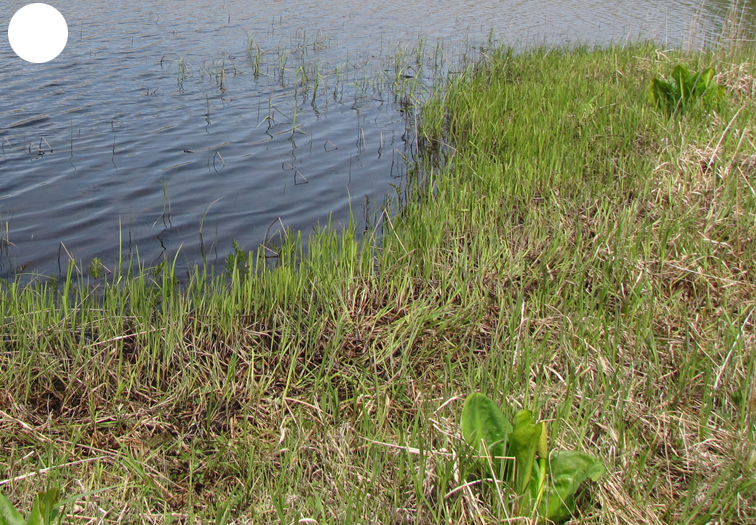
Tanfil'ev Island, sedge-moss bog

**Table 1.** Species of ground beetles from Kunashir Island not included in the present study.

**Species**	**locality , date**	**collector**	**deposit**
*Agonumgracilipes* (Duftschmid, 1812)	vicinity of Yuzhno-Kurilsk, 2.VIII.1995	Yu. Marusik	FEB RAS
*Harpaluseous* Tschitschérine, 1901	env. Dubovoe, 11.IX.1976	V. Kuznetsov	FEB RAS
*Stenolophuscastaneipennis* Bates, 1873	Peshchanoe Lake, west coast, 17-18.VIII.1980	S. Storozhenko	FEB RAS
*Trichotichnusseptemtrionalis* (Habu, 1947)	vicinity of Yuzhno-Kurilsk, 2.VIII.1995	Yu. Marusik	FEB RAS

**Table 2.** Island areas and the number of ground beetle species in the southern Kuril Islands.

Island	Species number	Area, km^2^
Kunashir Isl.	168	1510.20
Shikotan Isl.	68	252.77
Zelyony Isl.	2	58.38
Tanfil’ev Isl.	21	12.42
Polonsky Isl.	27	11.78
Yuriy Isl.	23	9.98
Anuchina Isl.	1	1.96
